# Oral health in adults with coronary artery disease and its risk factors: a comparative study using the Korea National Health and Nutrition Examination Survey data

**DOI:** 10.1186/s12872-021-01878-x

**Published:** 2021-02-04

**Authors:** Sun Kyung Lee, Seon Young Hwang

**Affiliations:** 1 Graduate School of Hanyang University, Seoul, South Korea; 2 School of Nursing, Hanyang University, 222 Wangsimni-ro, Seondong-gu, Seoul, 04763 Korea

**Keywords:** Cardiovascular disease, Coronary artery disease, Oral health, Oral hygiene, Propensity score

## Abstract

**Background:**

This study aimed to examine the relationship between oral health status and hygiene behavior among adults and elderly with preceding chronic disease or coronary artery disease history.

**Methods:**

Data were obtained from the Korea National Health and Nutrition Examination survey conducted from 2016 to 2017. Cardiovascular risk group was defined as adults over the age of 30 with hypertension, diabetes, dyslipidemia, previous myocardial infarction, or angina. Adult and elderly groups were separated and analyzed by 1:1 propensity score matching (PSM), and complex sample logistic regression analysis was performed using SPSS and R programs.

**Results:**

In adults, 25.1% of healthy group and 41.9% of the risk group were diagnosed with periodontal disease by dentist. In the elderly, 40.0% of the risk group had chewing problems and 17.5% had speaking problems. After PSM, in adults (n = 1661 each), both univariate and multiple logistic regression analyzes showed that the prevalence of periodontal disease was significantly higher in the risk group than in the healthy group (Odds Ratio = 1.21, *p* = .028). In the elderly (n = 715 each), univariate analysis showed that the risk group had more chewing and speaking problems than the healthy group, but there was no difference in multivariate analysis.

**Conclusions:**

Adults under the age of 65 years with cardiovascular risk require periodic evaluation and education on the importance of maintaining oral health for primary or secondary prevention. Healthcare professionals should provide patient education to help them maintain adequate oral health and oral hygiene.

## Background

The association between periodontal diseases and chronic diseases is a cause for concern, and the rates of those affected with periodontal or chronic diseases continues to climb both in Korea and worldwide. According to the 6^th^Korea National Health and Nutrition Examination Survey (KNHANES) data, cardiovascular disease patients were 2.97 times more likely to have periodontal disease than those without cardiovascular disease [1]. Periodontal disease and loss of teeth increase the prevalence of chronic diseases such as hypertension, hyperlipidemia, stroke, myocardial infarction, and/or diabetes [2]. It has been reported that patients with periodontal disease were 1.38 times more likely to develop cardiovascular disease, after excluding patients who already had angina, myocardial infarction, or stroke [3].

These observations may occur because blood vessel atherosclerosis decreases the blood supply to periodontal tissues which impairs the ability to resist anaerobic bacteria based on oxygen concentration. As a result, periodontal disease may occur, eventually leading to tooth loss [4]. *P. gingivalis*, one of the major periodontal bacteria, induces platelet coagulation, invades the coronary and carotid endothelial cells, increases the possibility of macrophage infiltration, and causes endothelial dysfunction through stimulation of blistering cell conversion and atherosclerosis progression [5].

Periodontal disease such as periodontitis and dental caries is the second most common disorder among Korean adults. In 2016 alone, the increase among patients who received periodontal treatment on a yearly basis was13.3% [6]. The prevalence of periodontal disease is approximately 22.1% in Korean females and 31% in males; the prevalence of dental caries is also high (24.9% in females and 33.4% in males) [7]. Coupled with the 21.2% increase in medical examination fees for periodontal disease, there has been a noticeable decrease in people’s quality of life, increasing both individual and societal burden [8]. Similarly, more than a quarter of the global death rate (31.3%) is due to chronic cardiovascular disease [9, 10].

Death due to coronary artery disease (CAD) affects 31 males and 21 females for every 100,000 individuals in the Korean population—a number that has been continuously increasing in Korea over the last 10 years [11]. The prevalence of preceding chronic diseases among adults over the age of 30 are also on the rise, at 29.1% for hypertension, 26.5% for diabetes, and 19.9% for dyslipidemia [12]. A systematic literature review [13] suggested that reducing the incidence and prevalence of periodontal disease may reduce related systemic chronic diseases such as cardiovascular disease and diabetes.

Oral health hygiene practices—non-smoking, maintaining appropriate weight, proper toothbrushing, use of dental floss and interdental brushes, as well as regular exercise and periodic dental examinations—are required to promote periodontal health [14]. However, people with chronic diseases such as hypertension and osteoporosis have lower oral health behaviors than those without such diseases [15]. Sabounchi et al. [16] also reported on the significant negative effects of smoking and positive effects of physical exercise on periodontal health. In cohort follow-up study of 941 in-patients with CAD, cardiovascular disease recurrence was decreased by 81%in patients who used dental floss or interdental brushes [17]. Although causality between atherosclerosis and periodontal disease has not been confirmed, a close association is indicated through improvement of atherosclerosis with increased oral hygiene behavior such as flossing [18]. Therefore, attention must be paid to the oral health of patients with preceding chronic disease as primary prevention, as well as those with CAD requiring secondary prevention due to the possibility of recurrence.

As the prevalence of periodontal disease and tooth mortality with atrophy and recession of gum and dental tissues increase with age [19, 20], age must be considered in the study approach. Among lifestyle risk factors, cigarette smoking and low physical activity were associated with periodontal health, respectively [16]. Therefore, using national representative data, this study aimed to confirm the association of oral health and hygiene behavior in adults and the elderly with preceding chronic disease or CAD through comparison with a healthy group. Results can be used as basic data to confirm the importance of oral hygiene management for primary and secondary prevention in adults and the elderly with cardiovascular risk factors.

## Methods

### Research design

This was a secondary data analysis conducted using a cross-sectional correlational study design using data from the 7th KNHANES conducted in 2016 and 2017.

### Setting and sample

**Fig. 1:**
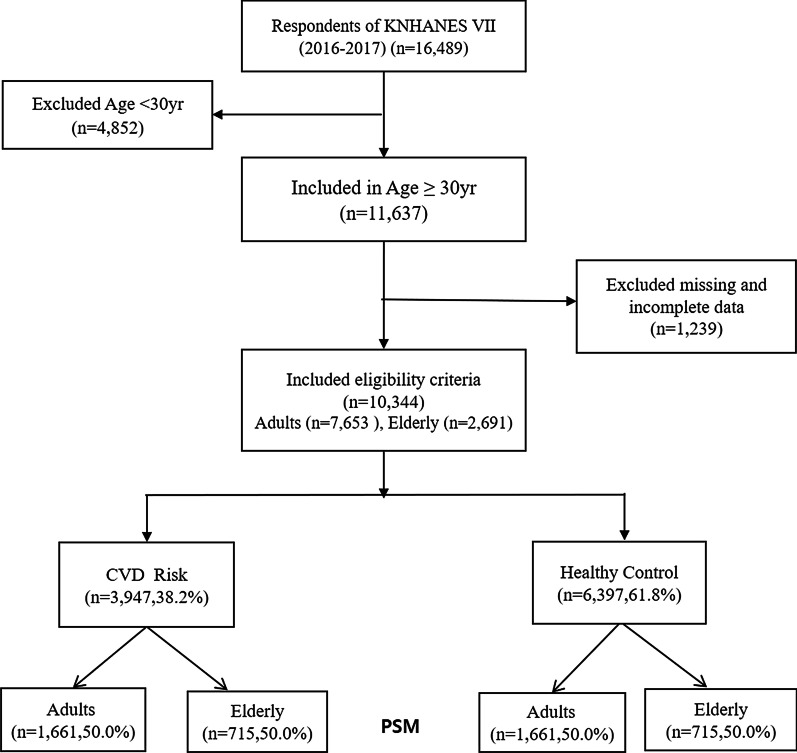
Flow chart of the study population. *CAD* coronary artery disease, *KNHANES* Korea National Health and Nutrition Examination survey, *PSM* propensity score matching

We used a nationally representative database obtained from the 7th KNHANES, a government-approved statistical survey by the Korean Centers for Disease Control and Prevention. The KNHANES includes data on participants’ demographic, social, health, and nutritional status using three component surveys: health interview, health examination, and nutrition survey. The samples included 16,489 participants from the 1st and 2nd years of the KNHANES raw data (2016–2017). The KNHANES sampling method included a two-stage stratified sampling approach with sampling districts and households as the first and second sampling units. A systematic sampling method in the 192 sampling areas was used in the selection of the 23 appropriate sample households. Facilities such as nursing homes, military facilities, prisons, and foreign households were excluded [21]. Among these, a total of 10,344 subjects were selected, excluding those who were under 30 years of age (n = 4852), persons with other chronic diseases such as cancer or stroke, and subjects with missing values, as they did not undergo dental examinations or did not provide any response to the oral health behavior questionnaire (n = 1239). Of the 10,344 patients, 38.2% (n = 3947) had a history of one or more of having hypertension, diabetes, dyslipidemia, acute myocardial infarction, or angina, and were classified as risk groups, and the remaining 61.8% were classified as healthy controls. As a result of matching the propensity score, in the adult group, 1661 people were in the risk group and the healthy group, respectively, and in the elderly group, 715 people were in the risk group and the healthy group (Fig. 1).

## Measurements

### PSM variables and clinical characters

The 10 sociodemographic and lifestyle-related characteristics were selected as variables for PSM in the risk group and control group based on the literature review. Selected sociodemographic variables used for PSM included age, sex, education level, household income, marital status, living area, employment, and family history of cardiovascular disease. For lifestyle-related characteristics, the selected variables were smoking and body mass index.

In this study, the risk group was considered to have been diagnosed with CAD such as myocardial infarction and angina in the past, or one of hypertension, diabetes, or dyslipidemia.

### Oral health status

Oral examination was performed by a trained public health dentist to confirm whether the subjects had periodontal disease and to ensure the reliability of the periodontal health survey.

In this study, the prevalence of periodontal disease was assigned as ‘0’ to ‘no’ and ‘1’ to ‘yes’, and those with one or more permanent tooth decay were classified as having caries. To determine the number of remaining teeth, 0 is healthy teeth, 1 is caries, 3 is caries-experience treatment, 4 is loss of caries-experienced, 6 is full-color teeth, 7 is caries-non-experienced treatment, and 8 is unerupted teeth, 9 is a non-recordable surface to investigate the condition of each tooth. 4, 5, and 8 were changed to 0 as a case of no teeth, and the remaining 0, 1, 3, 6, 7, 9 were changed to 1, and a total of 32 teeth were summed and calculated. The number of remaining teeth was divided into 20 or more and less than 20, which are the number of possible mastication in consideration of functional aspects [22].

Oral health status consisted of dental hospital use, simple dental treatment, dental nerve treatment, gingival disease treatment, tooth extraction or oral surgery experience, number of remaining teeth, permanent dental caries, prevalence of periodontal disease and subjective responses to chewing and speaking difficulties over the past year. The subjective responses on chewing issues were based on a 5-item scale response to the question “Do you experience discomfort when chewing food due to intraoral problems such as teeth, dentures, and gums?” Answers stating “Extremely uncomfortable” and “Uncomfortable” were reclassified as “Yes”, while answers of “So-so”, “Not uncomfortable”, and “Not at all uncomfortable” were reclassified as “No” for the analysis. The subjective response on speech issues was also based on a five-item scale response to the question “Do you experience discomfort in accurate pronunciation due to intraoral problems with the teeth, dentures, or gums?” Answers of “Extremely uncomfortable” and “Uncomfortable” were reclassified as “Yes” while answers of “So-so”, “Not uncomfortable”, and “Not at all uncomfortable” were reclassified as “No” for the analysis.

### Oral hygiene behavior

Five questions were used to assess oral hygiene behavior. Use of oral hygiene supplies was classified as “Yes” and “No” based on the use of dental floss, interdental brush, mouth rinse, electric toothbrush, and other products (tongue cleaner, water pick, hi-tech toothbrush). Daily toothbrushing less than three times and more than three times were reclassified as “No” and “Yes”, respectively, for the analysis. Preventive dental treatment was reclassified as “Yes” if sealants, fluoride application, or scaling were received, and “No” if not. The respondents were classified as “Yes” and “No” depending on whether they underwent a preventive oral examination to check their oral health status.

### Statistical analyses

Statistical analysis was conducted with the statistical packages SPSS 21.0 (IBM SPSS Statistics, Chicago, IL, USA) and R program (https://www.r-project.org); the PSM was conducted with PSM variables (age, sex, education level, household income, marital status, family medical history, residential area, occupation status, smoking status, body mass index), and a complex sample analysis was conducted to identify the differences. Moreover, in order to look at the differences in the oral health status, oral health behavior, treatment history, number of remaining teeth, permanent dental caries, and periodontal disease prevalence in the samples obtained via PSM, complex samples general linear model and chi-square test were conducted. Finally, complex sample logistic regression analysis was conducted to identify factors related to patients with cardiovascular risk.

## Results

### Sample characteristics before PSM in adults and the elderly

Before PSM matching, the characteristics of the subjects in the adult and elderly groups were analyzed. In the adult groups, differences in the demographic characteristics between the risk group and controls showed that the control group (45.4 years old) was younger than the risk group (54.6 years old). The education level in the risk group was lower than that in the control group, and the household income was higher in the control group than in the patient group. More participants in the control group were single/divorced/bereaved than those in the patient group, and more patients had a family history than those in the control group. Concerning obesity, the percentage of people with body mass index ≥ 25 kg/m^2^ was higher in the risk group than in the control group. After PSM, there was no difference in PSM variables (age, sex, education level, household income, marital status, family medical history, residential area, occupation status, smoking status, body mass index) between the risk group and controls.

In the elderly group, differences in the demographic characteristics between the risk group and controls showed that the control group was younger with an average age of 71.1 years compared to 72.8 years. There was no difference in the education level between groups, with greater lower middle-class people in the risk group compared to the control group. There was no difference in the marital status between the two groups, and more patients had a family history compared to the controls. There was no difference in the smoking status between the two groups, and the percentage of people with a body mass index ≥ 25 kg/m^2^ was higher in the risk group than in the control group. After PSM was conducted, there was no difference in the PSM variables (age, sex, education level, household income, marital status, family medical history, residential area, occupation status, smoking status, body mass index) between the risk and controls in the elderly group (Tables 1, 2).

**Table 1:** Baseline characteristics of the risk and control groups in adults (age < 65)

Variables	Categories	Unadjusted data			Propensity-matched data		
		Control (n = 5639), M ± SD, n (%)	At risk (n = 2014), M ± SD, n (%)	*p*	Control (n = 1661), M ± SD, n (%)	At risk (n = 1661), M ± SD, n (%)	*p*
Age (years)		45.41 ± 0.19	54.60 ± 0.20	< .001	53.78 ± 0.22	53.42 ± 0.22	.205
Sex	Male	2273 (38.0)	990 (46.4)	< .001	818 (46.4)	784 (43.7)	.165
	Female	3366 (62.0)	1024 (53.6)		843 (53.6)	877 (56.3)	
Education	≤ Middle school	689 (12.3)	692 (34.9)	< .001	467 (28.0)	507 (31.4)	.095
	≥ High school	4950 (87.7)	1322 (65.1)		1194 (72.0)	1154 (68.6)	
Household income	Middle-low	1680 (29.4)	786 (37.9)	< .001	599 (35.4)	602 (35.6)	.902
	Middle-high	3959 (70.6)	1228 (62.1)		1062 (64.6)	1059 (64.4)	
Marital status	Married	5040 (89.5)	1913 (95.8)	< .001	78 (95.8)	1569 (95.5)	.687
	Single/others	599 (10.5)	101 (4.2)		572 (4.2)	92 (4.5)	
Family history of CVD	No	2307 (41.8)	613 (29.9)	< .001	1583 (35.0)	548 (32.3)	.175
	Yes	3332 (58.2)	1401 (70.1)		1089 (65.0)	1113 (67.7)	
Living area	Urban	4846 (86.7)	1615 (82.4)	.002	291 (15.9)	297 (16.1)	.887
	Rural	793 (13.3)	399 (17.6)		1370 (84.1)	1364 (83.9)	
Employment	None/housewife	1490 (28.1)	585 (30.2)	.154	447 (29.3)	490 (30.9)	.412
	Employed	4149 (71.9)	1429 (69.8)		1214 (70.7)	1171 (69.1)	
Smoking	Never/ex-smoker	4518 (80.9)	1566 (79.5)	.281	1282 (78.4)	1272 (78.8)	.819
	Current smoker	1121 (19.1)	448 (20.5)		379 (21.6)	389 (21.2)	
BMI	< 25 kg/m^2^	3932 (71.1)	1050 (53.6)	< .001	976 (59.5)	940 (58.0)	.435
	≥ 25 kg/m^2^	1707 (28.9)	964 (46.4)		685 (40.5)	721 (42.0)	

*CVD* cardiovascular disease, *BMI* body mass index

**Table 2:** Baseline characteristics of the risk and control groups in the elderly (age ≥ 65)

Variables	Categories	Unadjusted data			Propensity-matched data		
		Control (n = 758), M ± SD, n (%)	At risk (n = 1933), M ± SD, n (%)	*p*	Control (n = 715), M ± SD, n (%)	At risk (n = 715), M ± SD, n (%)	*p*
Age	71.09 ± 0.182	72.78 ± 0.16	< .001	71.32 ± 0.19	71.18 ± 0.22	.623	
Sex	Male	355 (45.0)	771 (39.4)	.017	327 (43.9)	360 (49.7)	.050
	Female	403 (55.0)	1162 (60.6)		388 (56.1)	355 (50.3)	
Education level	≤ Middle school	522 (67.7)	1418 (71.9)	.070	495 (68.1)	490 (69.0)	.763
	≥ High school	236 (32.3)	515 (28.1)		220 (31.9)	225 (31.0)	
Household income	Middle-low	524 (67.6)	1472 (75.4)	.001	497 (68.1)	493 (68.7)	.817
	Middle-high	234 (32.4)	461 (24.6)		218 (31.9)	222 (31.3)	
Marital status	Married	754 (99.5)	1916 (99.1)	.217	711 (99.5)	710 (99.3)	.595
	Single/others	4 (.5)	17 (.9)		4 (.5)	5 (.7)	
Family history of CVD	No	537 (69.9)	1069 (53.8)	< .001	495 (68.2)	498 (68.3)	.973
	Yes	221(30.1)	864 (46.2)		220 (31.8)	217 (31.7)	
Living area	Urban	586 (82.1)	1462 (79.2)	.138	160 (17.9)	183 (21.8)	.077
	Rural	172 (17.9)	471 (20.8)		555 (82.1)	532 (78.2)	
Employment	None/housewife	451 (59.2)	1339 (71.8)	< .001	438 (61.0)	452 (66.5)	.057
	Employed	307 (40.8)	594 (28.2)		277 (39.0)	263 (33.5)	
Smoking	Never/ex-smoker	701 (91.8)	1787 (92.6)	.550	665 (92.7)	645 (90.6)	.198
	Current smoker	57 (8.2)	146 (7.4)		50 (7.3)	70 (9.4)	
BMI	< 25 kg/m^2^	556 (75.3)	1096 (57.0)	< .001	514 (73.9)	485 (68.5)	.069
	≥ 25 kg/m^2^	202 (24.7)	837 (43.0)		201 (26.1)	230 (31.5)	

*CVD* cardiovascular disease, *BMI* body mass index

### Oral health status and oral hygiene behavior in adults and the elderly after PSM

As a result of conducting PSM, in adults, 41.0% of the risk group reported of periodontal disease prevalence, showing a statistical significance compared to 36.2% of the control group (*p* = 0.017). No other characteristics regarding oral health status, oral hygiene behavior such as the use of oral products, dental examinations, toothbrushing frequency, and preventative treatment, treatment history, number of remaining teeth, permanent dental caries were significantly different in the two adult groups.

In elderly group, 40.6% of the risk group reported of a chewing problems, showing a statistical significance compared to 34.8% of the elderly control group (*p* = 0.039). Also, 18.7% of the risk group reported of a speaking problem, showing a statistical significance compared to 13.2% of the elderly control group (*p* = 0.013). No other characteristics regarding oral hygiene behavior such as the use of oral products, dental examinations, toothbrushing frequency, preventative treatment, treatment history and number of remaining teeth, permanent dental caries, and periodontal disease prevalence were significantly different in the two elderly groups (Tables 3, 4).

**Table 3:** Comparison of oral health between the risk and control groups before and after propensity score matching in adults (age < 65 years)

Variables	Unadjusted data			Propensity-matched data		
	Control (n = 5639), n (%)	At risk (n = 2014), n (%)	*p*	Control (n = 1661), n (%)	At risk (n = 1661), n (%)	*p*
Chewing problem						
No	4802 (85.3)	1492 (75.5)	< .001	1289 (77.7)	1246 (76.4)	.496
Yes	837 (14.7)	522 (24.5)		372 (22.3)	415 (23.6)	
Speaking problem						
No	5442 (96.5)	1848 (92.4)	< .001	1548 (93.4)	1534 (93.1)	.775
Yes	197 (3.5)	166 (7.6)		113 (6.6)	127 (6.9)	
Use of oral hygiene supplies						
No	2044 (36.0)	916 (44.4)	< .001	708 (41.5)	741 (43.7)	.285
Yes	3595 (64.0)	1098 (55.6)		953 (58.5)	920 (56.3)	
Oral examination in the last 1 year						
No	3334 (58.9)	1200 (59.5)	.688	984 (59.9)	979 (58.9)	.638
Yes	2305 (41.1)	814 (40.5)		677 (40.1)	682 (41.1)	
Tooth brushing per day						
< 3 times	2273 (39.8)	1004 (48.8)	< .001	788 (45.5)	808 (47.6)	.311
≥ 3 times	3366 (60.2)	1010 (51.2)		873 (54.5)	853 (52.4)	
Preventive dental treatment^a^						
No	3658 (64.2)	1244 (61.2)	.048	1072 (63.8)	1031 (61.5)	.251
Yes	1981 (35.8)	770 (38.8)		589 (36.2)	630 (38.5)	
Dental hospital use						
No	2425 (42.9)	773 (38.2)	.002	664 (40.7)	646 (38.8)	.337
Yes	3214 (57.1)	1241 (61.8)		997 (59.3)	1015 (61.2)	
Simple dental treatment^a^						
No	4679 (83.2)	1671 (83.0)	.871	1389 (84.1)	1379 (83.0)	.462
Yes	960 (16.8)	343 (17.0)		272 (15.9)	282 (17.0)	
Dental nerve treatment^a^						
No	4951 (88.1)	1676 (82.9)	< .001	1395 (84.8)	1387 (83.5)	.373
Yes	688 (11.9)	338 (17.1)		266 (15.2)	274 (16.5)	
Gingival disease treatment^a^						
No	4935 (87.8)	1619 (80.9)	< .001	1366 (82.8)	1328 (80.8)	.181
Yes	704 (12.2)	395 (19.1)		295 (17.2)	333 (19.2)	
Tooth extraction or oral surgery^a^						
No	5216 (92.8)	1796 (89.0)	< .001	1495 (90.2)	1479 (89.1)	.405
Yes	423 (7.2)	218 (11.0)		166 (9.8)	182 (10.9)	
Number of remaining teeth						
< 20	364 (6.6)	328 (15.4)	< .001	230 (13.8)	240 (13.2)	.658
≥ 20	5275 (93.4)	1686 (84.6)		1431 (86.2)	1421 (86.8)	
Permanent dental caries patients						
No	3989 (72.0)	1454 (73.1)	.425	1201 (74.6)	1199 (73.2)	.427
Yes	1650 (28.0)	560 (26.9)		460 (25.4)	462 (26.8)	
Prevalence of periodontal disease						
No	4178 (74.9)	1153 (58.1)	< .001	1033 (63.8)	965 (59.0)	.017
Yes	1461 (25.1)	861 (41.9)		628 (36.2)	696 (41.0)	

^a^Responses were based on experience within the previous year

**Table 4:** Comparison of oral health between the risk and control groups before and after propensity score matching in the elderly (age ≥ 65 years)

Variables	Unadjusted data			Propensity-matched data		
	Control (n = 758), n (%)	At risk (n = 1933), n (%)	*p*	Control (n = 715), n (%)	At risk (n = 715), n (%)	*p*
Chewing problem						
No	486 (64.5)	1125 (60.0)	.061	461 (65.2)	423 (59.4)	.039
Yes	272 (35.5)	808 (40.0)		254 (34.8)	292 (40.6)	
Speaking problem						
No	654 (87.1)	1580 (82.5)	.014	615 (86.8)	583 (81.3)	.013
Yes	104 (12.9)	353 (17.5)		100 (13.2)	132 (18.7)	
Use of oral hygiene supplies						
No	461 (60.8)	1259 (64.9)	.071	435 (61.1)	442 (60.5)	.840
Yes	297 (39.2)	674 (35.1)		280 (38.9)	273 (39.5)	
Oral examination in the last 1 year						
No	543 (69.5)	1418 (73.2)	.115	509 (69.4)	519 (73.3)	.174
Yes	215 (30.5)	515 (26.8)		206 (30.6)	196 (26.7)	
Tooth brushing per day						
< 3 times	447 (56.9)	1228 (62.7)	.014	418 (56.3)	439 (60.6)	.150
≥ 3 times	311 (43.1)	705 (37.3)		297 (43.7)	276 (39.4)	
Preventive dental treatment^a^						
No	525 (67.0)	1389 (71.1)	.066	493 (66.9)	502 (70.3)	.222
Yes	233 (33.0)	544 (28.9)		222 (33.1)	213 (29.7)	
Dental hospital use						
No	305 (38.8)	731 (39.0)	.925	286 (38.8)	272 (39.2)	.901
Yes	453 (61.2)	1202 (61.0)		429 (61.2)	443 (60.8)	
Simple dental treatment^a^						
No	664 (86.3)	1726 (89.8)	.038	631 (87.1)	634 (89.6)	.211
Yes	94 (13.7)	207 (10.2)		84 (12.9)	81 (10.4)	
Dental nerve treatment^a^						
No	623 (81.4)	1622 (83.7)	.218	590 (82.1)	602 (84.0)	.366
Yes	135 (18.6)	311 (16.3)		125 (17.9)	113 (16.0)	
Gingival disease treatment^a^						
No	625 (81.3)	1572 (81.7)	.855	590 (81.5)	570 (79.3)	.346
Yes	133 (18.7)	361 (18.3)		125 (18.5)	145 (20.7)	
Tooth extraction or oral surgery^a^						
No	653 (85.0)	1631 (84.6)	.793	620 (85.9)	589 (82.3)	.099
Yes	105 (15.0)	302 (15.4)		95 (14.1)	126 (17.7)	
Number of remaining teeth						
< 20	345 (43.7)	958 (50.1)	.012	331 (44.3)	342 (49.2)	.097
≥ 20	413 (56.3)	975 (49.9)		384 (55.7)	373 (50.8)	
Permanent dental caries patients						
No	556 (73.8)	1435 (75.4)	.439	524 (73.8)	534 (75.7)	.456
Yes	202 (26.2)	498 (24.6)		191 (26.2)	181 (24.3)	
Prevalence of periodontal disease						
No	417 (54.9)	992 (51.8)	.239	392 (54.7)	359 (51.2)	.251
Yes	341 (45.1)	941 (48.2)		323 (45.3)	356 (48.8)	

^a^Responses were based on experience within the previous year

The logistic regression analyses of the matched data showed that the prevalence of periodontitis in adults under the age of 65 was found to be a predictor that increased the risk of cardiovascular disease by 1.21 (95% confidence interval, 1.020–1.436) times compared to the group without periodontitis (Table 5).

**Table 5:** Adjusted logistic regression analyses on propensity-matched data in adults and elderly

Variables	Adults (< 65 years)		Elderly (≥ 65 years)	
	Odds ratio (95% CI)	*p*	Odds ratio (95% CI)	*p*
Chewing problem				
No	1		1	
Yes	1.039 (.833–1.297)	.732	1.083 (.833–1.408)	.552
Speaking problem				
No	1		1	
Yes	1.007 (.719–1.412)	.966	1.346 (.929–1.948)	.116
Use of oral hygiene supplies				
No	1		1	
Yes	.928 (.780–1.104)	.398	1.122 (.861–1.463)	.392
Oral examination in the last 1 year				
No	1		1	
Yes	1.028 (.846–1.249)	.781	.852 (.621–1.169)	.320
Tooth brushing per day				
< 3 times	1		1	
≥ 3 times	.935 (.794–1.101)	.418	.867 (.681–1.105)	.248
Preventive dental treatment				
No	1		1	
Yes	1.117 (.908–1.375)	.293	.899 (.650–1.244)	.519
Dental hospital use				
No	1		1	
Yes	.993 (.771–1.280)	.960	1.053 (.744–1.490)	.769
Simple dental treatment				
No	1		1	
Yes	1.042 (.805–1.348)	.755	.870 (.574–1.317)	.509
Dental nerve treatment				
No	1		1	
Yes	1.023 (.797–1.313)	.860	.793 (.567–1.109)	.174
Gingival disease treatment				
No	1		1	
Yes	1.073 (.863–1.334)	.524	1.158 (.835–1.604)	.378
Tooth extraction or oral surgery				
No	1		1	
Yes	1.048 (.788–1.395)	.746	1.327 (.924–1.905)	.125
Number of remaining teeth				
< 20	1		1	
≥ 20	1.116 (.865–1.440)	.396	.927 (.726–1.185)	.546
Permanent dental caries patients				
No	1		1	
Yes	1.058 (.881–1.272)	.543	.837 (.645–1.086)	.180
Prevalence of periodontal disease				
No	1		1	
Yes	1.210 (1.020–1.436)	.028	1.161 (.911–1.480)	.228

## Discussion

In this study, more differences in lifestyle and oral health-related characteristics were identified between the adult risk and healthy control groups than in the elderly groups before PSM. In adults younger than the age of 65, the risk was significantly higher among those who smoking and were obese compared to that in the healthy control group. This is consistent with the results of a previous study that, when demographic characteristics and health status were adjusted, the risk of periodontitis was higher if the body mass index increased, smoking, failure to use oral care products, and no dental check-up [23].

Smoking was found to negatively affect periodontal health, whereas physical exercise was reported to positively affect periodontal health [16]. Based on these results, the risk group should modify their lifestyle, with emphasis on smoking cessation and exercise to prevent periodontal and cardiovascular diseases. However, in adults, the risk group had significantly higher periodontal disease prevalence compared to those in the healthy control group. Compared to the healthy control group, the risk group also showed low oral hygiene behaviors in using hygiene supplies, daily toothbrushing, annual preventive dental treatment, and dentist visits. Poor oral hygiene has a significant impact on oral health, and can lead to variety of problems such as periodontitis, tooth decay, tooth or gum pain and discomfort, tooth infection and loss of teeth. Also, it can lead to complications such as swallowing, chewing, and speech difficulties [24]. These findings support the results of the 2012 KNHANES data showing that the need for periodontal disease treatment is high among smokers, the obese, and those lacking physical activity [25]. It can thus be inferred that lifestyle habits are an important factor affecting oral health status. As such, those in the cardiovascular risk group, particularly adults under 65 years old, should practice healthy lifestyle habits to improve their oral health.

After PSM, 41.0% of the adult risk group had significantly higher likelihood of periodontal disease prevalence compared with the healthy control group 36.2%. This finding was similar to the results of Baek et al. [26], which comprised 785 adults with metabolic syndrome. As a result of logistic regression analysis, the risk of periodontitis was 1.67 times higher in the case of metabolic syndrome compared to the normal case. It was demonstrated that person having MS had significantly higher odds of having periodontitis than normal people [26]. However, in the elderly, subjective feelings such as discomfort during speaking and during chewing did not differ between risk and healthy control groups in this study. These findings indicate that speech discomfort caused by oral problems, especially in adults, predicts cardiovascular risk and should be followed by early dental checkups and oral care.

Experience of gingival treatment related to oral bacterial infection was significantly higher in the adult risk group after PSM than in the healthy control group. This suggests that there is a higher frequency of dental problems such as periodontitis in the risk group that received more dental treatment. Shetty et al. [27] reported that gum disease treatment reduced the risk of heart disease and improved the health outcome of patients with periodontal disease and vascular heart problems. Accordingly, it is necessary to improve education and awareness about dental treatment and preventive management in the adult population with cardiovascular risk rather than the elderly population.

After PSM, the experience rate of tooth extraction or oral surgery among the oral health status variables was significantly higher in the risk group than in the healthy control group in the elderly. This was supported by the results of Lu et al.’s study [28] of 13,527 Chinese adults who had their teeth removed and subsequently experienced high frequency of complications such as hypertension, CAD, stroke, diabetes, and arrhythmia. Similarly, Lee et al. [29] reported that the risk of tooth loss occurred due to inappropriate oral hygiene practices such as poor toothbrushing and non-usage of oral hygiene products. Moreover, the chewing problem among the oral health status variables was significantly higher in the risk group than in the healthy control group in the elderly as well as the speaking problem, the risk group was 18.7%, higher than the healthy control group 13.2%. These results showed similar results to other previous studies. According to the results of previous studies, chewing and speaking problem in the elderly can act as a factor that worsens overall health. The elderly people are exposed to chronic diseases, and most of them are reported as factors that aggravate periodontal disease. Therefore, it is considered necessary to change the perception of not only dental workers but also medical workers in mutual cooperation and establish a mutual linkage system. However, there was no significant difference in oral hygiene behavior across all adult and elderly groups in the current study. Instead, the reason for the lack of difference between the risk and control groups in oral hygiene behavior could be attributed to the inclusion of lifestyle factors, such as exercise, as a variable for PSM.

While oral hygiene behavior is important in oral health and preventing cardiovascular disease, it has yet to be confirmed whether it can be a risk group predictor for both adult and elderly subjects after PSM. In the raw data prior to PSM, oral hygiene behavior including daily toothbrushing frequency, use of oral hygiene supplies, and preventive dental visits in the adult risk group was significantly worse than that in the control group. However, in the elderly, there were no significant differences between the two groups for hygiene behaviors. Most hygiene behaviors were also worse than those of adult subjects. A study of the National Health Screening Cohort in 247,696 healthy adults aged 40 years and over without a history of cardiovascular disease reported that toothbrushing more than once a day and periodic dental checkups reduced the risk of cardiovascular disease by 9% and by 14%, respectively. In addition, improved oral hygiene behavior has been shown to prevent periodontal disease, tooth decay, and tooth loss, as well as reduce the risk of cardiovascular disease [30]. This suggests that oral hygiene behavior such as frequent toothbrushing and regular dental visits reduced the risk of future cardiovascular events in healthy adults.

This study has several limitations. As patients were limited to those with preceding chronic disease and CAD, the results cannot be extrapolated to patients with other chronic diseases. It should also be noted that as cross-sectional research based on the 7th KNHANES (2016–2017), the focus was on understanding the relationship between variables rather than explaining causal relationships. Future studies will thus require longitudinal clinical studies and interventional studies to determine whether periodontal treatment can prevent cardiovascular disease. Moreover, further research on the variables to confirm the effectiveness of cardiovascular disease prevention in the practice of oral health and oral hygiene behavior is also necessary.

## Conclusions

This study was conducted to determine the association between oral health status, hygiene behavior, and cardiovascular risk including preceding chronic diseases in adults and the elderly, respectively, when sociodemographic and lifestyle-related factors were controlled. The adult risk group had significantly higher likelihood of periodontal disease prevalence compared with the healthy control group. On the other hand, chewing and speaking problem were found to significantly higher in elderly risk group than in the healthy control group. Consequently, healthcare providers should pay attention to their oral health status to prevent cardiovascular disease in risk group with preceding chronic diseases or CAD, especially adults, and help them to conduct regular oral examinations and periodontal disease management.

## Data Availability

All information was made public, and the data were downloaded from the KNHANES homepage (http://knhanes.cdc.go.kr) after reviewing the “Regulation on the disclosure and use of source data of the National Health and Nutrition Survey.”

## Abbreviations

CAD: Coronary artery disease
KNHANES: Korea National Health and Nutrition Examination Survey
KCDC: Korea Centers for Disease Control and Prevention
PSM: Propensity score matching
CVD: Cardiovascular disease
BMI: Body mass index
